# Insect pathogenic fungi and bed bugs: behaviour, horizontal transfer and the potential contribution to IPM solutions

**DOI:** 10.1007/s10340-017-0943-z

**Published:** 2017-12-13

**Authors:** Anders Aak, Morten Hage, Bjørn Arne Rukke

**Affiliations:** Department of Pest Control, Norwegian Institute of Public Health, Lovisenberggata 8, Postboks 4404, 0456 Nydalen, Oslo, Norway

**Keywords:** *Cimex lectularius*, Integrated pest management (IPM), *Beauveria bassiana*, Horizontal transfer, Aggregation, Activity

## Abstract

The increasing problem of bed bugs requires the development of new control strategies, and insect pathogenic fungi can contribute towards management. We used laboratory bioassays with *Isaria fumosoroseus*, *Lecanicillium muscarium* and *Beauveria bassiana* to evaluate their virulence to the bed bug. Only *B. bassiana* significantly affected bed bug survival and was dependent on dose and formulation. A 2% *B. bassiana* oil formulation induced horizontal transfer to elevate mortality in a 10-day arena bioassay. Temporal distribution of contagious individuals and increasing the dose from 2 to 4% did not increase mortality. Horizontal transfer mainly occurred between adults, and only partly between adults and nymphs. Bed bugs showed activity peaks during the night, and activity was increased by elevated levels of CO_2_. Distribution between harbourages was not affected by CO_2_ activation, level of infection or the bio-pesticide, and horizontal transfer was not dependent on the degree of aggregation. Movement in the arenas negatively affected horizontal transfer when the number of susceptible individuals was large. Level of infection also influenced behaviour as the bed bug movement increased with elevated disease burden. The use of fungi as a part of an integrated pest management strategy seems to be an interesting option that should be investigated further. *B. bassiana* kills bed bugs and can be carried to harbourages to target hidden individuals.

## Key message

- Insect pathogenic fungi may contribute in control strategies against bed bugs.
- *Beauveria bassiana* significantly affected bed bug survival and was dependent on dose and formulation.
- Horizontal transfer mainly occurred between adults.
- As a part of an IPM strategy, the consistent mortality and the horizontal transfer may contribute to elevated population mortality and improved control by reaching hidden or passive individuals.

## Introduction

The worldwide return of the blood feeding bed bugs (*Cimex lectularius*, Hemiptera: Cimicidae) is a result of pesticide resistance, increased travel, ineffective control, trade with second-hand furniture and unawareness of preventive measures in the accommodation industry and among the general public (Doggett et al. 2018 in press). The difficulty of eradication can largely be assigned to pesticide resistance (Dang et al. 2017; Davies et al. 2012) and a cryptic and nocturnal lifestyle of the bed bugs (Reinhardt and Siva-Jothy 2007). This has caused an increased focus on developing control strategies and promoted the use of integrated pest management (IPM), where new and partly experimental tools such as trapping (Olson et al. 2017; Singh et al. 2013; 2015), steam treatment (Loudon 2017; Puckett et al. 2012) and desiccant dusts (Aak et al. 2016; Akhtar and Isman 2016; Benoit et al. 2009; Singh et al. 2016; Wang et al. 2013) can supplement more conventional approaches (Doggett et al. 2012; Koganemaru and Miller 2013).

Biological control against bed bugs is not commonly accepted as an expedient method, but insect pathogenic fungi may contribute to bed bug management strategies. The *Beauveria* and *Metarhizium* genera can kill a wide range of immature and adult insects in eco-agricultural systems (Faria and Wraight 2007; Hajek 2004), and adaption for indoor use against bed bugs seems possible. *Beauveria bassiana* (Hypocreales, Cordycipitaceae) has been reported as a suitable fungus (Barbarin et al. 2012, 2017), while *Metarhizium anisopliae* (Hypocreales, Clavicipitaceae) has in laboratory studies been found effective only under moist conditions (Ulrich et al. 2014) and consequently is suspected to have limited effect in relatively dry urban environments such as human bed rooms. In addition to mortality from direct exposure, it is also promising that fungal spores are horizontally transferred from exposed to unexposed individuals in sufficient amounts to kill the insects (Barbarin et al. 2012). Conversely, suspected anti-fungal properties (inhibition of growth and elastase production) of the aggregation chemicals, (E)-2-octenal and (E)-2-hexenal, contribute to uncertainty with respect to true efficiency (Battinelli et al. 2006; Ulrich et al. 2015). To date, studies on fungi and bed bugs have only been conducted in small petri dish-sized containers. This offers limited movement possibilities and hardly any opportunities for natural interactions connected to host searching and aggregation activities (Barbarin et al. 2012, 2017; Ulrich et al. 2014, 2015). Insect–fungi interactions that impact behaviour, disease severity or conidia dissemination have been shown in many insects, including blood feeders (Dimbi et al. 2009; Garza-Hernández et al. 2015; Quesada-Moraga et al. 2004; Roy et al. 2006; Ugine et al. 2014), and more elaborate study designs should therefore be used to obtain a better understanding of functionality under semi-natural conditions to elucidate the potential benefits and limitations of insect pathogenic fungi as a bed bug control method.

Disease severity and infection spread are in general regulated by multiple causes and are likely to be promoted by a high population density, a large number of contagious individuals, frequent subject contact and exchange of individuals between sub-populations (Bellows and Hassel 1999; Birkemoe et al. 2016; Rothman 2012; Rukke et al. 2011). Bed bugs spend most of their time in aggregations (Pfiester et al. 2009; Reinhardt and Siva-Jothy 2007), where thigmotaxis (stop-response to touch stimulus) ensures prolonged and direct contact with other bed bugs (Olson et al. 2009), and there are abundant interactions between individuals (Reinhardt and Siva-Jothy 2007). Bed bugs also have traumatic insemination (Siva-Jothy 2006), including attempts at homosexual and nymphal mating (Harraca et al. 2010; Ryne 2009), and individuals move between different aggregation sites (Cooper et al. 2015; Wang et al. 2010). Bed bugs mostly leave their harbourages for feeding and return after ingesting their blood meal (Aak et al. 2014; Reis and Miller 2011; Suchy and Lewis 2011). This dynamic situation provides an opportunity to develop a system where spores are obtained during questing (movements outside harbourages) and disseminated to other individuals within harbourages (hiding places). Questing activity and aggregation is regulated by semiochemicals such as CO_2_ and harbourage odours, in combination with the physiological state of the bed bugs (Aak et al. 2014; Gries et al. 2015; Olson et al. 2017; Reis and Miller 2011; Ulrich et al. 2016; Weeks et al. 2013) and is likely to act together with biological properties of the fungi to impact control efficiency.

The magnitude of transmission within the population following inundation application in a biocontrol strategy is unknown, but we anticipated that the interplay between bed bug behaviour and horizontal transfer can contribute to overall virulence. Consequently, we performed laboratory bioassays in closed small-scale systems to reveal the virulence of two commercially available products, which were then used in arena bioassays that represented control situations with variable predefined infection levels and a simulated presence of a host. This allowed us to describe and connect the CO_2_-initiated host search and bed bug aggregation levels with inoculated dose, infection rate, disease development and horizontal transfer in semi-natural bed bug populations.

## Materials and methods

### Insects

Bed bugs in the stock cultures were sampled from two hotels in Oslo, Norway, in 2009, and all experimental animals were fed heated human blood through a Parafilm membrane (Aak and Rukke 2014). To produce uniform experimental animals, fourth and fifth instar nymphs were selected from the stock cultures and provided with a blood meal. Newly hatched adults and nymphs emerged after 10–14 days and less than 1 week prior to the start of the experiment. Bed bugs were either kept in the arenas described below or in standardized 140-mL experimental polyethylene boxes with a ventilated lid (Aak and Rukke 2014). Adults were always fed before being used in the experiments, and all treatments maintained a balanced ratio between males and females. After feeding, males and females were kept together at 22 °C for 48 h in climate chambers (Sanyo MLR-351H, Medinor ASA, Oslo, Norway) with 16-h light/8-h dark cycles and 60% relative humidity before being exposed to the fungus or released into the arenas. Experiments took place in laboratories with equal light cycles, at 22–23 °C and a relative humidity of 40–50%.

### Product virulence

#### Products

Four different products were tested (Table 1). PreFeRal WG (*Isaria fumosorosea* strain Apopka 97, 2 × 10^9^ cfu/g, Biobest N.V., Belgium) and Mycotal (*Lecanicillium muscarium*, 1 × 10^10^ spores/g, Koppert BV, the Netherlands) was chosen as representatives for species that have not been tested against bed bugs, and BotaniGard 22WP (*Beauveria bassiana* strain GHA, 2 × 10^13^ cfu/kg, Laverlam International, USA) was chosen as a water-based product with a species known to cause mortality in bed bugs. These three products were prepared according to the manufacturer’s instructions by dissolving the product in water and diluting to the recommended concentration. Aprehend (*Beauveria bassiana* strain GHA, 2% spore suspension in oil, ConidioTec, USA) was included because it is a bed bug adapted ready-to-use product delivered by the manufacturer. Spore growth was measured according to the growth protocol for water or oil preparations (Oliveira et al. 2015), and germination percentages were found to be within 58–78% as indicated as the expected range by the producers.

**Table 1:** Fungi and product properties of bio-pesticides tested in a closed system, with persistent exposure of adult bed bugs (*Cimex lectularius*) to inoculated substrates

Product	Fungus	% conidia	Solvent	Amount applied on substrate (g)	% germination	Conidia/cm^2^	Conidia/cm^2^ (adjusted for sporulation)	Substrate
BotaniGard	*B. bassiana*	0.02	Water	1.12	64	6.9 × 10^5^	4.2 × 10^5^	Cloth
Aprehend	*B. bassiana*	2.00	Oil	1.02	78	5.3 × 10^7^	4.1 × 10^7^	Cloth
PreFeRal	*I. fumosoroseus*	0.1	Water	1.29	68	8.4 × 10^4^	5.1 × 10^4^	Cloth
Mycotal	*L. muscarium*	0.1	Water	1.15	58	3.7 × 10^5^	2.2 × 10^5^	Cloth

#### Exposure to conidia

The substrate used was circular pieces of cotton cloth with diameters of 47 mm (woven bed sheets, 100% cotton, Jysk—Oslo, Norway), which were impregnated with conidia by dipping them in the suspension (Table 1). The spore-covered substrates were air-dried in a ventilated laboratory for 3–4 days (Mycotal, PreFeRal and BotaniGard) or 10–12 days (Aprehend) until surface dry. The conidia-loaded substrate was then placed on the bottom of the experimental boxes, and the slippery polyethylene ensured that the bed bugs grabbed hold of the cloth and stayed on it for the duration of the exposure. All four products were tested by using eight boxes with six bed bugs in each box. An additional eight boxes without fungi were used as controls for Mycotal, PreFeRal and BotaniGard, and eight boxes with bed bugs placed on cloth pieces dipped in the oil without conidia acted as controls for Aprehend.

#### Mortality assessment

Mortality was recorded daily by blowing gently into the polyethylene boxes. Bed bugs waving their antennae, shifting stance or moving were considered alive. Exposure was terminated after 10 days, and any survivors were transferred to new boxes with a clean filter paper as substrate. These individuals were checked for mortality on day 17 and day 24. Animals that died during the experiments were immediately removed and checked for mycosis by individual drying over silica gel for 1 week before incubation in moist chambers and visually assessing the fungal colonization and sporulation.

### Population studies in arenas

#### Experimental protocol

**Fig. 1:**
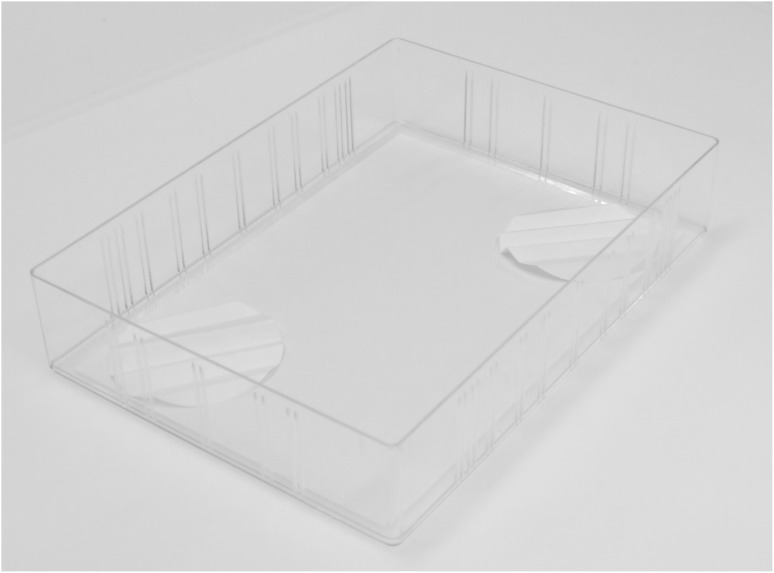
Plastic arena (31 cm × 22 cm × 6 cm) with paper bottoms used for simulation of bed bug (*Cimex lectularius*) control situations with fungi (*Beauveria bassiana*). Harbourages of 9 cm width were made from accordion-folded circular filter papers

To simulate a bed bug control situation with two distinct aggregations of bed bugs, we used arenas with two available harbourages made from accordion-folded circular filter papers (qualitative filter paper – ø = 90 mm, VWR, Oslo, Norway), placed diagonally across from each other (Fig. 1). The arenas were constructed from hard plastic transparent boxes (31 cm × 22 cm × 6 cm, Ultra-Plast A4/60, VWR, Norway), and a 29 cm × 20 cm self-adhesive paper (Herma GmbH, Filderstadt, Germany) was attached to the bottom of the box to provide grip for the crawling bed bugs. Newly emerged adults were selected, fed and left to rest for 48 h on filter paper in polyethylene boxes, together with newly emerged unfed fifth instar nymphs. We always used 12 adults and 8 nymphs in each arena, and the required proportion of uninfected adult animals and all nymphs were released into the centre of the arenas. During the first hour of the experiment, we infected the remaining adults by allowing them to walk on cloth with spores (prepared as described above), before releasing them into the centre of the arena. Bed bugs were kept in the arena for 10 days without interference to allow natural interactions to occur and infection to spread within the population. The laboratory had installed 3 cameras and lights needed for night and day video recording of bed bug movements in 18 arenas simultaneously (Aak et al. 2016), and the entire 10-day experiment was video-recorded. After 10 days, adults and nymphs were collected, mortality was registered, and survivors were transferred to polyethylene boxes with a filter paper. These individuals were checked for delayed mortality on day 17 and day 24. The numbers of faecal spots and eggs deposited in each of the two harbourages were registered to provide a measure of distribution. Bed bugs that died during the 24 days of experimentation were checked for mycosis, as well as all survivors.

#### Experiments

Based on results from the initial test, expected fast mortality was achieved by using Aprehend, and slow mortality was achieved with BotaniGard. A total of 108 arena replicates were equally divided between the two fungal formulations.

To investigate the combined efficiency of direct infection and horizontal transfer, we used 72 arena replicates. We infected either 33, 67 or 100% of the adults at the start of the experiments, and to evaluate the effect of induced questing activity with suspected increased intermingling of individuals and possible reduced contact time inside harbourages, CO_2_ was added to half of the arena replicates. CO_2_-producing units made from a 5 L plastic can containing 3 L tap water, 0.5 kg table sugar and 5.0 g yeast (La-Hem super yeast, Mariestad, Sweden), were used to simulate human presence. The cans were prepared approximately 24 h before use, and they produced approximately 50 mL CO_2_ per minute. Three stimulant canisters were included on nights 1 and 2 and nights 5 and 6 of the experiment, and they were left inside the arena room for 6 h.

To simulate a contagion situation with dual introduction of fungal carriers into the bed bug populations with initial infection rates of 33, 67 or 100%, we used an additional 36 arena replicates without CO_2_ stimulation. Half of the fungal carriers were exposed to spores and released from the start, and the remaining fungal carriers released after 5 days of the experiment. The last bed bugs to be released were kept in separate boxes next to the arena before being exposed to conidia on day 5.

To investigate the effect of increased conidia density on horizontal disease transmission, the arena experiments with a 33% infection level and 2% conidia dose were repeated with a 4% conidia dose in an additional 12 arena replicates. The experiment was performed with and without CO_2_ stimulation.

Twelve arenas with uninfected individuals (6 with CO_2_ stimulation and 6 without) were used as a control.

### Statistical methods and calculations

#### Statistical analyses

Analyses were performed using SigmaPlot 13.0 (Systat Software, San Jose, CA, USA) and JMP Pro 13.0.0 (SAS institute, Cary, NC, USA). Data were checked for normality, and pairwise comparisons were performed using the *t* test or paired t tests and multiple comparisons using ANOVA. If tests for normality failed, we used the nonparametric alternatives Mann–Whitney rank sum, Wilcoxon signed rank and Kruskal–Wallis ANOVA. The Kaplan–Meier product limit method was used with the log-rank test between groups to investigate survival, and the relationship between disease and behaviour was investigated by linear regressions with horizontal transfer as the dependent variable, and activity or aggregation as explanatory variables. The level of significance was set to 0.05 for all tests.

#### Horizontal transfer

The horizontal transfer in each arena was determined by the total number of individuals killed by fungi minus the number of initially exposed individuals. This number was divided by the number of susceptible individuals present at the start of the experiment in order to provide a proportional measure for surplus infection.

#### Aggregation

Most of the eggs (92%) were deposited inside harbourages. Both the number of eggs deposited, and the faecal spots in harbourages were used to calculate relative dwelling in the two harbourages, with a score ranging from complete aggregation (100:0 = 1.0) when all eggs or spots were found in one harbourage, to an equal distribution (50:50 = 0.0) when eggs or spots were equally distributed between the two harbourages. Eggs and spots were correlated (Pearson product-moment correlation *r*(264) = 0.738, *P* < 0.001), and the average of the two scores was used to connect the aggregation level with fungi spread in populations.

#### Activity

At every 30 min of the video recording, a 10-s sequence was observed. The number of individuals moving during these 10 s was used to describe night and day movements within each arena. Overall averages for all 10-s periods for the first 3 nights were used to connect arena activity with horizontal transfer.

## Results

### Product virulence

**Fig. 2:**
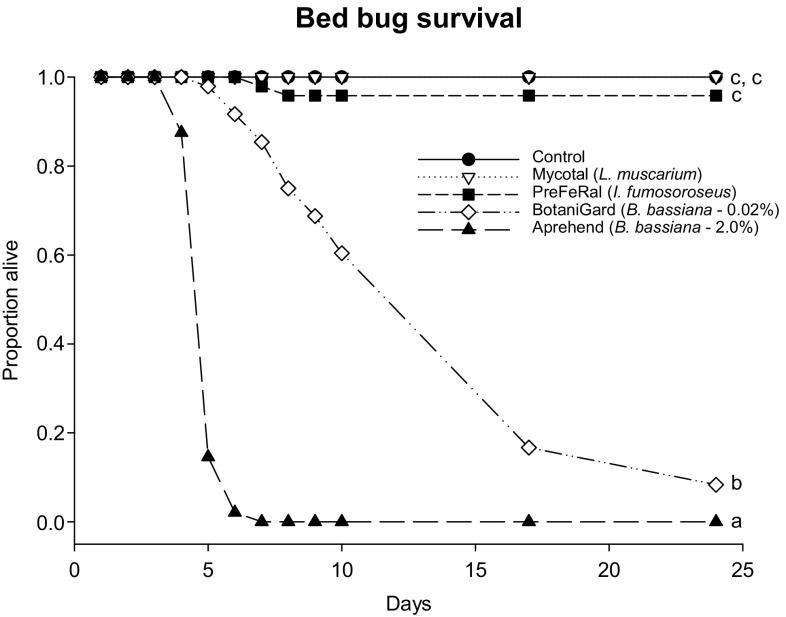
Bed bug (*Cimex lectularius*) survival after exposure to four different products with three different fungus species. Survival was tested on 48 adults per fungus, and bed bugs were exposed through cloth inoculated with conidia for the initial 10 days of the experiment. Different letters (a, b and c) denote significant differences (*P* < 0.05)

The control treatments experienced no mortality, and neither PreFeRal or Mycotal caused significant bed bug mortality relative to this control (Kaplan–Meier log-rank test; PreFeRal vs. control: *χ* ^2^ = 0.00, *P* = 1.0_no mortality_ and Mycotal vs. control: *χ* ^2^ = 2.02, *P* = 0.155, Fig. 2). BotaniGard and Aprehend significantly reduced survival compared to the control (Kaplan–Meier log-rank test; BotaniGard vs. control: *χ* ^2^ = 83.59, *P* < 0.001 and Aprehend vs. control: *χ* ^2^ = 109.77, *P* < 0.001, Fig. 2). Aprehend induced faster mortality compared to BotaniGard (Kaplan–Meier log-rank test; Apprehend vs. BotaniGard: *χ* ^2^ = 75.45, *P* < 0.001, Fig. 2).

### Population studies in arenas

**Fig. 3:**
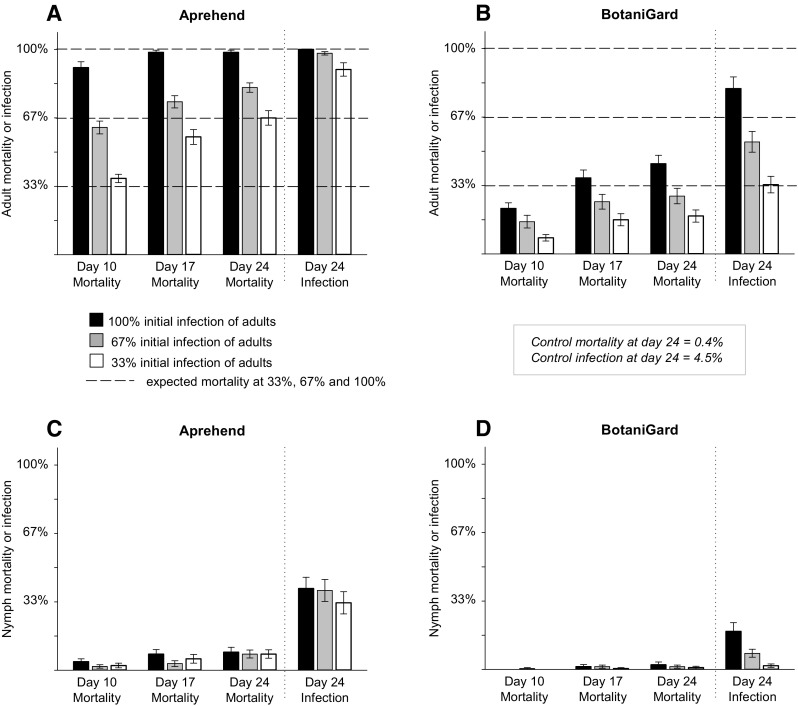
Accumulated average (± SE) bed bug (*Cimex lectularius*) mortality and fungal infection at day 10, 17 and 24 in experimental populations with 33, 67 and 100% of the adults being exposed to conidia (*Beauveria bassiana*) inoculated substrates for 1 h at the start of the experiment. Individual graphs show adult (**a**) and nymph (**c**) mortality/infection when using Aprehend (2% conidia in oil), and adult (**b**) and nymph (**d**) mortality/infection when using a BotaniGard (0.02% conidia in water)

A single bed bug died in the control arenas (0.4% mortality) without showing any sign of fungal growth, whereas all fatalities in the different contagion experiments were confirmed to be caused by fungi. Across all experimental treatments, Aprehend caused more than twice the number of mortalities compared to BotaniGard (paired *t* test 10, 17, 24 days; *t* = 18.31, *P* < 0.001, Fig. 3a vs. 3b). In populations with individuals infected with Aprehend, the anticipated adult mortality levels of 33, 67 and 100% were reached at day 10, and we detected efficient horizontal transfer when mortality exceeded the initial infection after 17 and 24 days. In populations with individuals infected with BotaniGard, the anticipated mortality was not reached in either of the treatments, and horizontal transfer was therefore not registered (Fig. 3b). Nymph mortality induced by horizontal transfer was low using both products and did not reach more than 11% (Fig. 3c, d). Distribution of contagious individuals across two infection events did not significantly affect mortality compared to simultaneous release (paired *t* test; *t* = 0.44, *P* = 0.67), and increasing the dose from 2 to 4% in the oil formulation did not elevate mortality (paired *t* test; *t* = 0.48, *P* = 0.65).

**Fig. 4:**
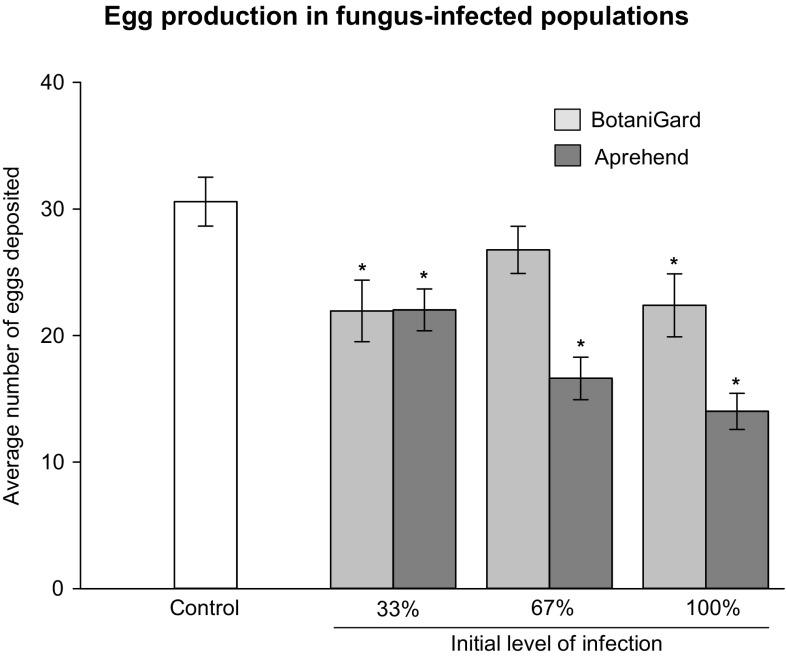
Average (± SE) egg production in bed bug (*Cimex lectularius*) populations suffering from fungal infections (*Beauveria bassiana*) induced by BotaniGard (0.02% conidia in water) or Aprehend (2% conidia in oil). The populations initially contained six adult females. Significant differences (*P* < 0.05) relative to the control are denoted by asterisk

There was an overall bed bug distribution of 81 and 19% between the two harbourages. This distribution was persistent across treatments and was not influenced by the presence or absence of CO_2_ stimulation (Mann–Whitney rank sum test: *T* = 3373.0, *P* = 0.393), level of infection (Kruskal–Wallis ANOVA: *H* = 6.9 *P* = 0.073) or the type of fungus used (Mann–Whitney rank sum test: *T* = 3293.5, *P* = 0.891). In experiments where horizontal transfer to adults could be observed, i.e. 2 and 4% conidia concentrations at 33 or 66% initial infections, the aggregation ratio ranged from 99 and 1% to 54 and 46%. No relationship between horizontal transfer and aggregation was observed among adults (linear regression: mortality *R* ^2^ = 0.04, *F* = 2.11, *P* = 0.15, infection; *R* ^2^ = 0.05, *F* = 2.42, *P* = 0.13) or nymphs (linear regression: mortality *R* ^2^ = 0.00, *F* = 0.17, *P* = 0.685, infection; *R* ^2^ = 0.04, *F* = 1.98, *P* = 0.17). Eggs deposited during the experiment were significantly influenced by fungal infections (ANOVA: *F* = 6.75, *P* < 0.001, Fig. 4). When compared to the controls, five out of six treatments were negatively affected, and Aprehend showed a decreasing egg production with increased levels of initial infection, in line with the observed mortality in the arenas.

**Fig. 5:**
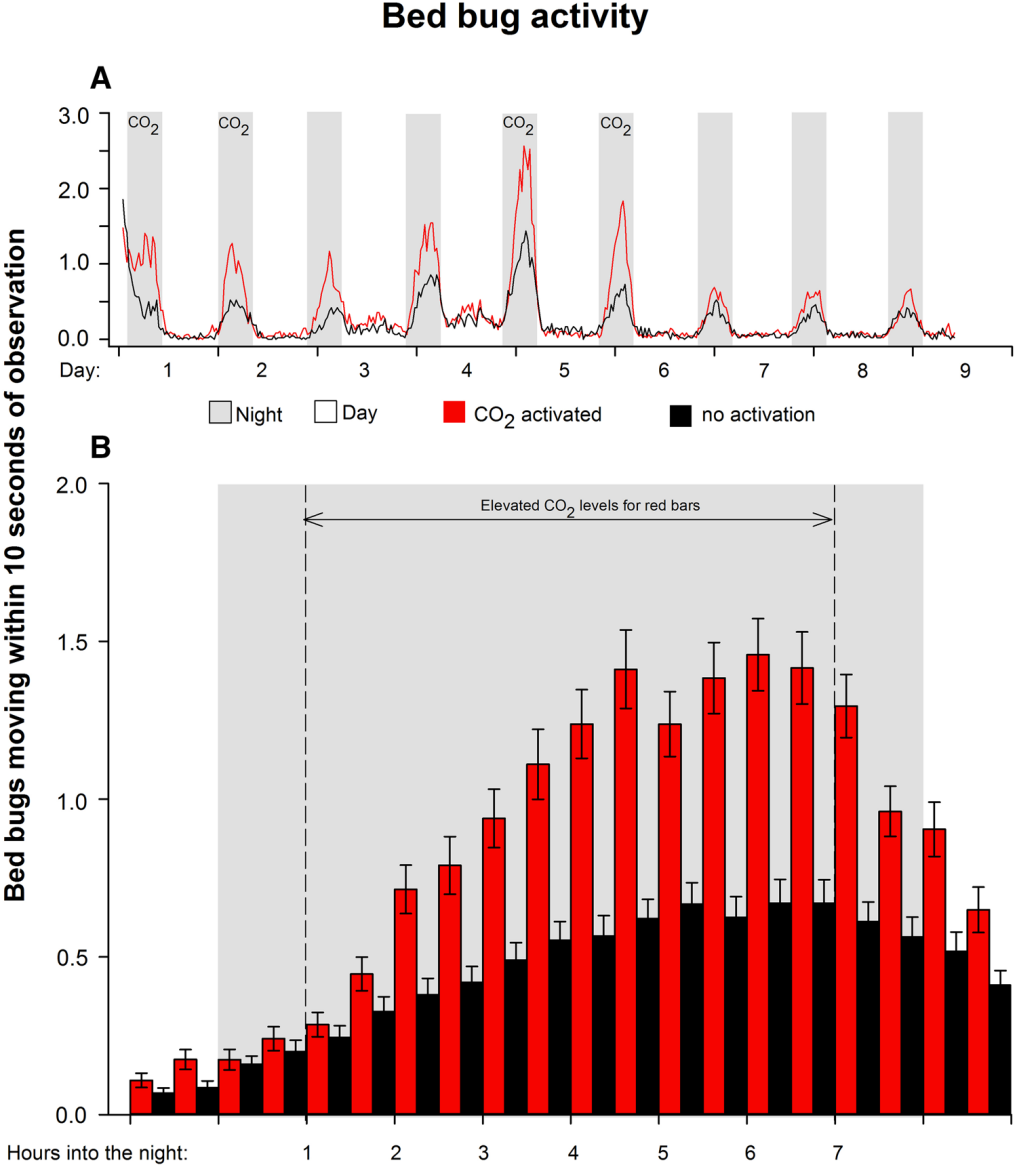
Bed bug (*Cimex lectularius*) activity in experimental arenas simulating control situations using insect pathogenic fungi (*Beauveria bassiana*). White background colour indicates lights on (day) and grey background colour indicates light off (night). CO_2_ levels were elevated by yeast fermentation of a sugar solution releasing approximately 150 mL/min for 6 h during the night (**b**) on experimental day 1, 2, 5 and 6 (**a**)

**Fig. 6:**
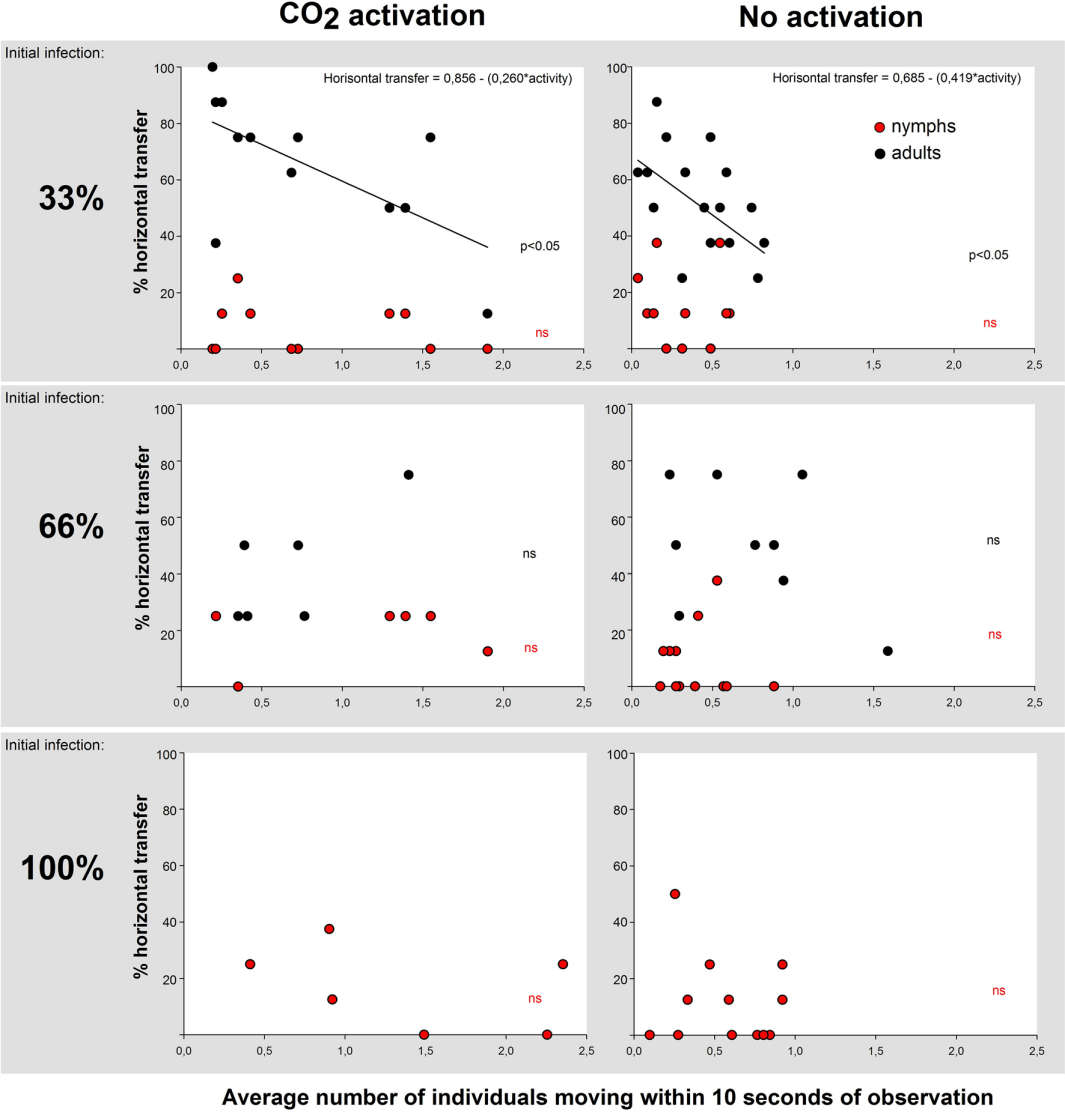
Horizontal transfer plotted against activity in experimental arenas simulating control situations using insect pathogenic fungi (*Beauveria bassiana*) to decimate bed bug (*Cimex lectularius*) populations. Populations had an initial adult infection of 33, 67 or 100%, and each population (*n* = 6, 12 or 18) contained 12 adults with a balanced sex ratio and 8 nymphs

**Fig. 7:**
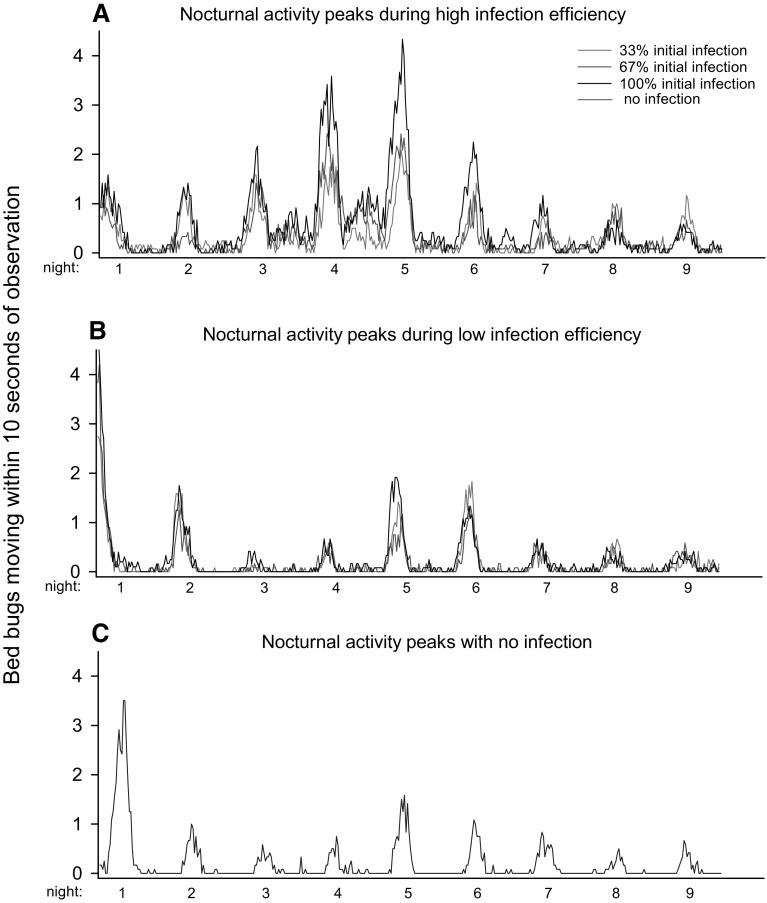
Activity in experimental arenas simulating control situations using insect pathogenic fungi (*Beauveria bassiana*) to decimate bed bug (*Cimex lectularius*) populations. Initial adult infection levels were 33, 67 or 100%, and the individual graphs show activity levels using Aprehend (2% conidia in oil) (**a**), BotaniGard (0.02% conidia in water) (**b**) and no infection (**c**)

Bed bugs showed activity peaks during the night and limited movements during the day (Fig. 5a). In addition to increased movement during nights with CO_2_ release, the two nights following the activation period maintained an elevated activity. Nocturnal peaks were reduced after night 6. During nights 1–6, when the differences between CO_2_-stimulated and CO_2_-unstimulated bed bugs were most pronounced, the activity was significant (paired *t* test; *t* = 6.84, *P* < 0.001, Fig. 5b), and peak activity was approximately 2.5 times higher in stimulated bed bugs compared to non-stimulated ones. Both activated and non-activated populations showed a gradual increase in activity lasting for 5–6 h into the night before declining towards the arrival of day (Fig. 5b). In populations having an initial infection ratio of 33%, we observed a significant relationship between horizontal transfer and activity. A higher level of activity reduced the horizontal transfer in both CO_2_-stimulated (linear regression: *R* ^2^ = 0.42, *F* = 7.12, *P* = 0.024) and CO_2_-unstimulated individuals (linear regression: *R* ^2^ = 0.22, *F* = 4.546, *P* = 0.049). At higher initial infection levels and among nymphs, there was no connection between movement and horizontal transfer (Fig. 6). The general activity (average of the three infection levels) also increased significantly with the disease burden (Wilcoxon signed rank test: *Z* = 13.94, *P* < 0.001, Fig. 7a vs. 7b, and *Z* = 2.42, *P* = 0.016, Fig. 7b vs. 7c), thus creating more restless populations close to mortality. This was particularly evident with Aprehend (high control efficiency), where activity also paralleled the infection levels around the expected time of death (majorities of deaths occurred after 4–6 days in the product virulence experiment). BotaniGard (low control efficiency) showed no consistent connection to infection levels apart from being elevated relative to the control.

## Discussion

Only one of the products was specifically adapted for killing bed bugs, and it was superior to the other tested products. This was likely due to the higher conidial concentrations and oil formulation benefits. A full screening, where formulation, dose, all relevant species and strains are accounted for, is needed for a complete demonstration of the potential of insect pathogenic fungi in bed bug control. However, the mortality appears strongly dependent on conidial concentration (BotaniGard—0.02% and Aprehend—2.00%) in both the product virulence test and in the population studies in the arenas. *B. bassiana* consequently seems promising as additional developments, product modifications and species-specific adaptions are likely to further improve efficiency (Lacey et al. 2015; Wang and Wang 2017). The results cannot be used to conclude the non-functionality of the other products, but the differences between *B. bassiana, I. fumosoroseus* and *L. muscarium*, at the three low doses, all dissolved in water, indicate that *B. bassiana* holds the highest potential for use as a bio-pesticide against bed bugs. *B. bassiana* is also the choice of species in the bed bug adapted product and our lack of improved control by doubling the dose may indicate that Aprehend’s 2% conidia solution is optimized according to bed bug behaviour. High conidial density also appears to be crucial for inducing horizontal transfer, capable of increasing population mortality, without the need for direct contact between bed bugs and the inoculated substrate. In the arena bioassays, we also observed a concurrence with expected behaviour, as questing mainly occurred during the night (Reis and Miller 2011; Romero et al. 2010), and increased with the presence of a host signal (Aak et al. 2014; Suchy and Lewis 2011). It is also likely that the free choice of harbourage, combined with thigmotaxis (Olson et al. 2009), produced a skewed aggregation that was strengthened by semiochemicals (Gries et al. 2015; Olson et al. 2017; Siljander et al. 2008; Ulrich et al. 2016) and bed bug movement between harbourages (Cooper et al. 2015). This natural order of conduct indicates authenticity of the bioassay, allowing extrapolation of results towards field applications.

The failure to find a connection between the aggregation level and horizontal transfer was surprising, because we assumed that increased aggregation would bring contagious and healthy individuals into closer proximity, thus promoting fungal infection. We cannot completely discard this hypothesis, as our experiments did not identify positioning or harbourage-to-harbourage movement of contagious individuals, and an equal relative distribution of infectors may balance horizontal transfer across aggregation levels. If males and females also search each other out for mating, the lack of connection with aggregation suggests that individual behaviour plays a more important role than spatial distribution. All our experimental populations had a balanced sex ratio, and we expect normal mating activity and limited trauma from repeated inseminations (Benoit et al. 2012; Kilpinen et al. 2012). However, mating activity is important for horizontal transfer in other insects (García-Munguía et al. 2011; Garza-Hernández et al. 2015; Peng et al. 2011; Quesada-Moraga et al. 2004; Reyes-Villanueva et al. 2011; Scholte et al. 2004; Toledo et al. 2007; Ugine et al. 2014), and the traumatic insemination of bed bugs is probably a key to elevated disease burden, as it promotes close individual contact and may let spores bypass the cuticle barrier (Reinhardt et al. 2005). This fits well with the observed differences between adults and nymphs and helps to explain the negative impact due to increased movement, since mating should be limited when adults are searching for a host. As observed among other blood feeding arthropods with comparable lifestyles, there may also be distinct differences in fungi efficacy or behaviour connected to life stage (Butt et al. 2016; Kirkland et al. 2004a, b; Wassermann et al. 2016) that may have played a role in affecting horizontal transfer and overall mortality across stages. A more speculative explanation relates to the anti-fungal properties of the aggregation volatiles (Ulrich et al. 2015). An elevated horizontal transfer of conidia in dense aggregations may have been masked by a parallel increase in anti-fungal secretions, but as the fungus appears to be highly functional in our experimental setting, these effects seem limited.

The study units with variable infection levels provide an insight into potential outcomes of *B. bassiana* treatment of bed bug-infested rooms. Considering that bed bug populations with only 33% of infected individuals resulted in twice as many fatalities, the potential of this control method appears to be good. As opposed to our fixed numbers of infective events and contagious individuals, a field population will gradually pick up new spores, which will replenish the conidia in the harbourages over time. The exact duration of *B. bassiana* horizontal transfer capabilities is unknown, but spores will either grow attached to the exoskeleton or lose their infective abilities. Flies and beetles infected with conidia often show a decline in potential conidia transfer during the initial days after exposure (Cárcamo et al. 2015; Peng et al. 2011; Toledo et al. 2007; Ugine et al. 2014). This creates a short transfer opportunity, but when considered in combination with the observed infection-induced stress, an interesting aspect regarding efficiency of transfer emerges. If the disease burden creates increasingly restless individuals, which are disengaged from natural aggregation and mating activities within just days of infection, the harbourage ratio of healthy to infectious individuals may shift in favour of disease carriers. When considered in combination with a conidia replenishment over time, this behavioural response may provide an escalation in fungus-induced mortality, as the population moves towards extermination.

The movement pattern of bed bugs with host signal responses (Aak et al. 2014; Suchy and Lewis 2011), and the intermingling of individuals between harbourages (Cooper et al. 2015) offer the potential to manipulate behaviour to optimize the impact from the fungi. To ensure that as many individuals as possible will come into contact with the conidia, it may prove valuable to promote the collection of spores by using a CO_2_ stimulant to increase activity in the treated room (Aak et al. 2016; Singh et al. 2013). The negative connection between activity and horizontal transfer highlights the need for an applied system to strike a balance between activity and rest, in order to benefit from horizontal transfer as well. Attract and infect is a strategy used to improve the delivery of biological control agents, by deploying attractive and inoculated devices to infect pest insects, which subsequently disseminate conidia into the population (Lacey et al. 2015). This approach also seems to fit the control of bed bugs, but since no strong attractant exists, a variation of the strategy could be to develop an activate-auto-disseminate approach, where both males and females cross-contaminate each other and infect healthy individuals. Manipulation of volatiles is easy in small accommodations like bedrooms (Aak et al. 2016), and there is therefore a potential low-cost improvement for bed bug control with insect pathogenic fungi.

Even though the killing capability appears promising, the time needed to drive a field population towards elimination by using insect pathogenic fungi is currently unknown. The total time required could in a worst-case scenario be as much as 30 days. As it is unlikely that spore application will reach the inside of all harbourages, and the horizontal transfer from adults to nymphs is low, the final instar nymphs taking a blood meal just before application will not be reached until after 5–10 days, when they have moulted and start questing. These newly emerged adults also need 5–10 days to succumb to the pathogens, and although infection reduced the egg production through increased mortality, we observed a large number of eggs in all arenas. These eggs will hatch to produce a new cohort, which needs an additional 5–10 days to be killed by the fungi. In such a worst-case scenario, it might also be possible for nymphs to moult and shed cuticle with conidia to avoid infection to delay or prevent full eradication. Additionally, all studies to date, including this one, have used forced conidia exposure although behavioural avoidance (Kaakeh et al. 1996; Kilpinen and Steenberg 2016; Meyling and Pell 2006; Ormond et al. 2011; Thompson et al. 2007) may impact field effects strongly. These uncertainties connected to efficiency may prove to be a major obstacle, as biting will persist for the duration of the treatment, and because the challenges connected to closing bed rooms or hotel rooms create the desire for a fast and efficient eradication. A related challenge is the dry indoor environment, which may reduce the overall efficiency of the fungi (Jaronski 2010; Lacey et al. 2015). This is unfortunate because the survival of a single female may lead to rebounding populations and consequently encourages the use of high conidia concentrations and possibly repeated applications. Although mostly harmless as an infectious agent in mammalian tissue (Zimmermann 2007), *B. bassiana* spore application in bedrooms clearly poses an elevated inhalation risk (Madsen 2011). It is well recognized that indoor fungal exposure is associated with the development or exacerbation of a variety of allergic and respiratory symptoms (Jaakkola et al. 2013; Kanchongkittiphon et al. 2015; Mendell et al. 2011). The potential health impacts of the treatment therefore need to be fully addressed to safeguard the residents’ health.

The use of fungi as part of bed bug control is an interesting option that should be pursued further on both an individual level and a population level. More knowledge connected to behaviour, behaviour-modifying factors, physiology, reproduction and senescence is needed for a greater understanding of the interactions between bed bugs and *B. bassiana* and, combined with application knowledge and field efficiency tests, it appears likely that fungi can contribute towards future bed bug control. The disadvantages connected to time and efficiency may be many when using fungi as a single method, but as a part of an IPM strategy, the consistent mortality and horizontal transfer may counteract pesticide resistance (Barbarin et al. 2017) and help in reaching hidden or passive individuals. This study indicates that fungi have the potential to utilize the natural bed bug biology to get carried to harbourages and by doing so, offer an elevated probability of control success, even without being the main contributor to population eradication.

## Author contributions

AA, MH and BAR conceived and designed the research. AA, MH and BAR conducted laboratory experiments. AA and BAR processed and analysed the data. All authors contributed to writing and approved the manuscript.
